# Simultaneous onset of visual dysfunction and cerebral infarction in a young patient with CADASIL: a case report

**DOI:** 10.3389/fmed.2026.1728053

**Published:** 2026-01-26

**Authors:** Guanlu Liang, Jiehui Xu, Zhenyu Wu

**Affiliations:** Department of Ophthalmology, Zhejiang Hospital, Hangzhou, Zhejiang, China

**Keywords:** CADASIL, case report, cerebral infarction, optic nerve/retinal ischemia, visual dysfunction

## Abstract

Cerebral autosomal dominant arteriopathy with subcortical infarcts and leukoencephalopathy (CADASIL) is a hereditary small arteriolar disease caused by mutations in the NOTCH3 gene. Acute vision loss is not commonly associated with the classical phenotype of CADASIL. We report a rare CADASIL case with the simultaneous onset of visual dysfunction and cerebral infarction in a young male. The patient was confirmed to have cerebral infarction on magnetic resonance imaging. Symptoms of acute vision loss occurred simultaneously due to optic nerve and retinal ischemia. Consequently, this case provides novel perspectives on the relationship between ocular hemodynamics and inherited cerebral small vessel disease. It is crucial to heighten awareness that presentation of non-arteritic anterior ischemic optic neuropathy (NAION) and retinal hypoperfusion in a young patient without any other risk factors necessitates consideration of secondary causes. These manifestations could represent a potential presentation of CADASIL.

## Introduction

Cerebral autosomal dominant arteriopathy with subcortical infarcts and leukoencephalopathy (CADASIL) is the most common hereditary cerebral small vessel disease. It is a progressive, monogenic disorder and was first recognized as a distinct entity in the early 1990s and genetically mapped to the NOTCH3 gene on chromosome 19q12 by Joutel et al. (1). It has 33 exons and codes for a transmembrane receptor protein of 2,321 amino acids, predominantly expressed in vascular smooth muscle cells (VSMCs) and pericytes (2, 3). It plays an important role in vascular development and function by regulating the proliferation, differentiation, maturation, migration, and apoptosis of VSMCs, as well as the proliferation of pericytes (4, 5).

The disease is characterized by recurrent ischemic strokes, progressive cognitive decline leading to vascular dementia, migraine with aura, and mood disturbances (6). Clinically, CADASIL presents with a highly variable but characteristic phenotype that typically manifests in mid-adulthood (between 30 and 50 years of age) (7), with variable severity even within the same family (8).

Diagnosis relies on a combination of clinical suspicion, family history, characteristic neuroimaging findings, and confirmatory genetic testing (9). Brain magnetic resonance imaging (MRI) is crucial, showing extensive, symmetric white matter hyperintensities (WMHs) on T2/FLAIR sequences in the periventricular and deep white matter, with characteristic early involvement of the anterior temporal poles and external capsules. Other markers include lacunar infarcts, cerebral microbleeds (on T2/SWI sequences), and enlarged perivascular spaces (10, 11).

Currently, there is no curative or disease-modifying therapy for CADASIL (12). Clinical management is entirely supportive and symptomatic. Early diagnosis and timely treatment are very important for prognosis.

Pathological hallmark is granular osmiophilic material (GOM) deposition in small arteries/arterioles, with vascular smooth muscle cell degeneration, impaired vasomotor function, and chronic subcortical hypoperfusion, leading to recurrent ischemia and tissue injury (6, 13).

In CADASIL patients, structural abnormalities in blood vessels may also involve ocular vasculature. However, it is noteworthy that acute vision loss due to ocular hypoperfusion is not a classic phenotype of CADASIL. This report presents the first case of simultaneous onset of visual dysfunction and cerebral infarction in a young CADASIL without typical vascular risk factors.

## Case presentation

**Figure 1:**
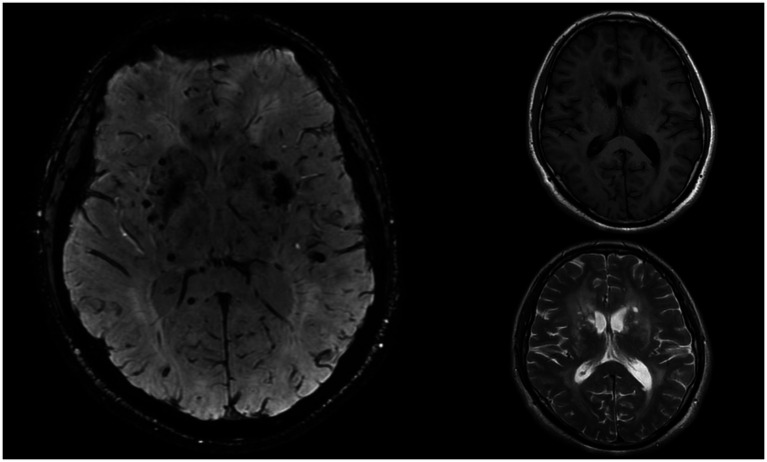
Cranial (SWI) reveals a hemorrhage in the left basal ganglia, along with diffuse multiple small hemorrhagic foci. Cranial MRI indicates a hemorrhagic focus and a recent infarct in the left basal ganglia region, scattered white matter hyperintensities (T2W), partial encephalomalacia foci, and subcortical arteriosclerotic encephalopathy.

**Figure 2:**
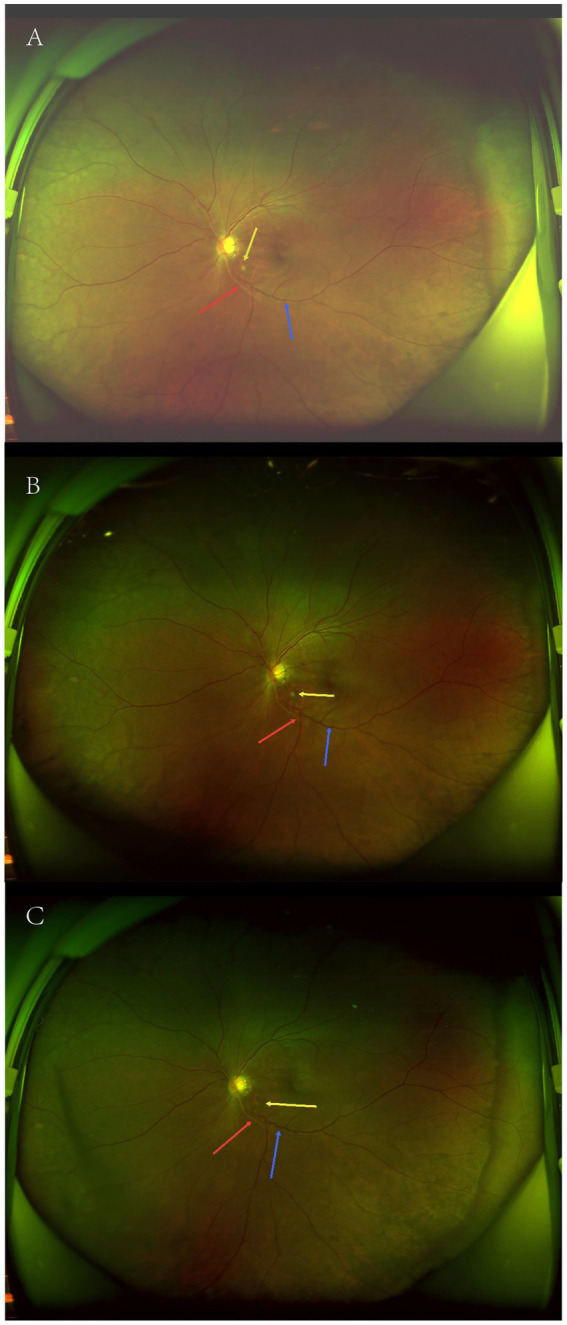
**(A)** Fundus photograph of the onset day. Fundus photography reveals blurred optic disc margins with reddish discoloration, tortuous veins (blue arrow), significantly attenuated arteries, particularly notable in the inferior retinal arteries (red arrow), and cotton wool spots (yellow arrow) observed at the inferotemporal region of the left optic disc. **(B)** Fundus photograph of the second day of follow-up blurred optic disc margins with reddish discoloration, tortuous veins (blue arrow), significantly attenuated arteries, particularly notable in the inferior retinal arteries (red arrow), and cotton wool spots (yellow arrow) observed at the inferotemporal region of the left optic disc. **(C)** Fundus photograph of 1-month follow-up. Tortuous veins (blue arrow), attenuated arteries show the arterial sheath (red arrow). After treatment, the cotton wool spots (yellow arrow) have improved significantly.

**Figure 3:**
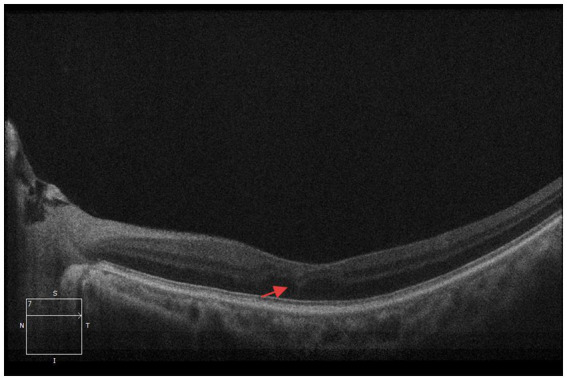
Optical coherence tomography (OCT) shows retinal ischemic perivascular lesions (RIPLs), which were characterized by a focal thinning of the inner nuclear layer, with an upward expansion of the outer nuclear layer (red arrow).

**Figure 4:**
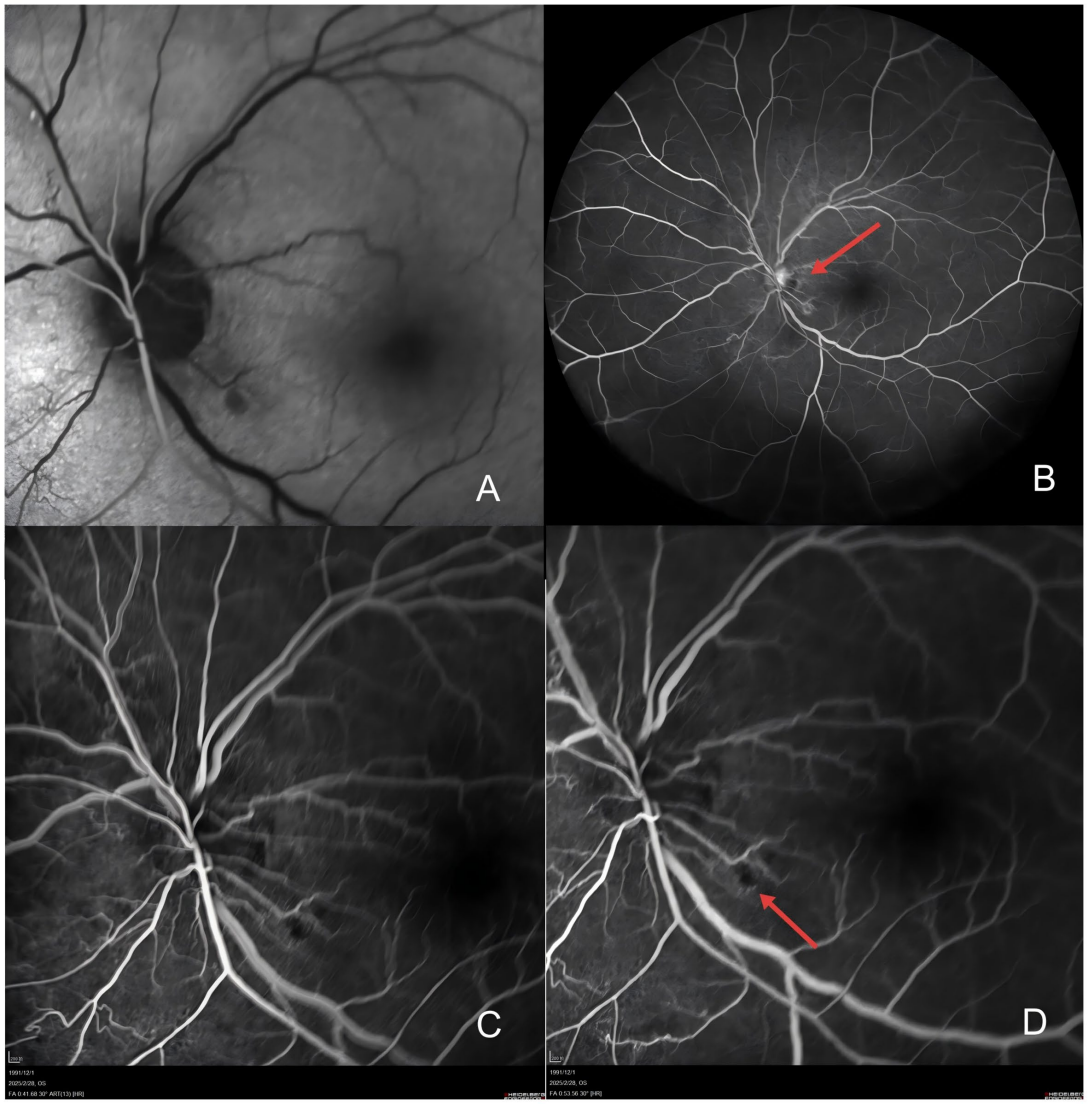
**(A)** Fundus fluorescein angiography (FFA) reveals left retinal arterial filling beginning at 26 s with markedly delayed perfusion. **(B)** FFA reveals an inferotemporal optic disc filling defect in the left eye. **(C)** FFA reveals left retinal venous filling was incomplete at 41 s. **(D)** FFA reveals left retina complete venous filling began at 53 s with markedly delayed perfusion and an ischemic lesion without perfusion in the inferotemporal part of the optic disc’s retina (red arrow).

A 33-year-old man was admitted to the department of neurology in our hospital due to slurred speech accompanied by right-hand weakness for 1 day. Cranial susceptibility-weighted imaging (SWI) revealed a hemorrhage in the left basal ganglia, along with diffuse multiple small hemorrhagic foci (chronic) in both cerebral hemispheres, cerebellar hemispheres, and the brainstem. Cranial MRI (plain scan + diffusion weighted imaging) indicated a hemorrhagic focus and a recent infarct in the left basal ganglia region, scattered white matter hyperintensities, partial encephalomalacia foci, and subcortical arteriosclerotic encephalopathy (Figure 1). Almost simultaneously, the patient developed blurred vision in the left eye. The vision loss was slight in the beginning, and he did not pay any attention. Three days later, he felt his vision suddenly deteriorated significantly, characterized by acute painless monocular visual loss. Ophthalmology consultation findings of left eye: visual acuity was hand motion (HM)/before eye (BE), transparent cornea, clear anterior chamber, sluggish pupillary light reflex in the left eye, relative afferent pupillary defect (RAPD) was positive, blurred optic disc margins with reddish discoloration, tortuous veins, significantly attenuated arteries, particularly notable in the inferior retinal arteries, and cotton wool spots observed in the inferotemporal region of the left optic disc (Figure 2A). Optical coherence tomography (OCT) showed retinal ischemic perivascular lesions (RIPLs), which were characterized by a characteristic focal thinning of the inner nuclear layer, with an upward expansion of the outer nuclear layer (Figure 3). Optical coherence tomography angiography (OCTA) revealed reduced macular vascular density, partly. The reduction of ganglion cells was also obvious. We gave the diagnosis: Left eye retinal arterial hypoperfusion, with suspected incomplete retinal arterial occlusion. We started the emergency management: anterior chamber paracentesis, ocular massage, sublingual nitroglycerin, oxygen therapy, topical brinzolamide to reduce intraocular pressure, and pentoxifylline for vasodilation. The follow-up (day 2) (Figure 2B): Visual acuity slightly improved to Fingers Counting (FC)/30 cm. Fundus fluorescein angiography (FFA) revealed left retinal arterial filling beginning at 26 s with markedly delayed perfusion (Figure 4A), incomplete retinal venous filling at 41 seconds (Figure 4C), complete venous filling at 53 s (Figure 4D). There was an ischemic lesion without perfusion in the inferotemporal part of the optic disc’s retina and an inferotemporal optic disc filling defect (Figure 4B). The diagnosis was left eye non-arteritic anterior ischemic optic neuropathy (NAION) and retinal arterial hypoperfusion. Treatment with compound thrombosis links agents (Fufang Xueshuantong, which was a type of traditional Chinese medicine, can promote the smoothness of blood vessels) and mecobalamin was initiated. The follow-up (1 month): Visual acuity was maintained 20/200. Left pupillary light reflex remained slightly sluggish, with persistent RAPD (+) showing improvement. Retinal veins were mildly tortuous, with attenuated arteries showing an arterial sheath. After the treatment, the cotton wool spots have improved significantly (Figure 2C). The patient underwent comprehensive tests, including blood lipid levels, blood glucose levels, erythrocyte sedimentation rate, computed tomography angiography (CTA) of the cervical vessels, B-ultrasound of the lower extremity arteries, and autoantibody tests. No significant abnormalities were found in any of these indicators. Given the patient’s young age, confirmed diffuse white matter hyperintensities, meaning cerebral infarction on MRI, absence of significant traditional risk factors or family history, combined with acute optic nerve and retinal ischemia, neurology was consulted for further etiological workup. They completed the genetic testing: genetic screening for hereditary cerebrovascular disorders identified a pathogenic NOTCH3 gene mutation, confirming a diagnosis of CADASIL.

## Discussion

Although CADASIL is not a commonly recognized disease affecting vision, preliminary studies have reported that a small subset of patients may experience transient ischemic attacks preceding stroke episodes or diagnosis (14). Research also identified characteristic funduscopic alterations in CADASIL patients even in the absence of overt ocular symptoms. These findings primarily include retinal arteriolar narrowing, arteriolar sheathing, increased arteriolar light reflex, and venous nicking, alongside retinal venous dilatation (15). However, patients do not exhibit clinically significant visual impairment. Furthermore, increasing capillaries on the surface of the retina has been observed in certain CADASIL cases, speculated to be associated with chronic hypoxia secondary to hemodynamic changes (16). Crucially, retinal vascular occlusions (both arterial and venous) and the presence of cotton wool spots are uncommon in CADASIL (15). The predominant vascular pathology observed in the retina, mirroring the cerebrovascular changes seen in CADASIL, is primarily characterized by a reduction in either blood flow volume or total retinal blood volume (17).

NAION is generally considered to result from vascular compromise of the optic nerve head and hemodynamic alterations leading to ischemic damage and subsequent visual loss. It predominantly affects middle-aged and elderly individuals, particularly those with underlying conditions such as hypertension and diabetes. When NAION presents in a young patient without typical vascular risk factors, secondary causes should be investigated, and CADASIL may be considered as one of the atypical etiologies (18).

In addition to NAION, macular changes also contributed to this patient’s visual impairment. There was a significant reduction in both macular vessel density and perfusion density on OCTA, which was consistent with the previous study (19). OCT revealed RIPLs, which were characteristic of focal thinning of the inner nuclear layer, with an upward expansion of the outer nuclear layer, along with the presence of cotton wool spots—a finding rarely reported in previous studies (20, 21). RIPLs represented an actionable imaging biomarker that can be harnessed to detect ischemia in the retina (22), which has been discovered and focused on in recent years. We hypothesized that these represented occlusions of small macular arterioles and infarctions of the nerve fiber layer, likely resulting from abnormal retinal capillary perfusion due to granular osmiophilic material (GOM) deposition in pericytes, leading to thickened arterial vessel walls and reduced retinal blood flow (23). CADASIL may cause a specific arteriopathy characterized by progressive degeneration and loss of VSMCs, thickening and occasional splitting of the arteriolar wall due to fibrosis and hyalinization, lumen stenosis, and occlusion of some arterioles (24, 25). VSMCs of CADASIL patients displayed increased proliferation and apoptosis, and cytoskeleton disorganization (26). This arteriopathy is systemic but mainly affects cerebral small to medium-sized penetrating arteries and leptomeningeal arteries. The most characteristic ultrastructural change in CADASIL is the GOM accumulating close to the membrane infoldings of VSMCs and pericytes (24, 25). GOM is periodic acid-Schiff (PAS)-positive, and also stains are eosinophilic or basophilic. Under electron microscopy (EM), GOM deposits are visible as particles of tightly aggregated and fine electron-dense granule materials with a size of 10–15 nm. In most CADASIL patients, GOMs are immunoreactive for NOTCH3 extracellular domain (NOTCH3^ECD^), suggesting NOTCH3^ECD^ is an important component of GOM. Therefore, the presence of GOM is characteristically present in the brains of patients with CADASIL.

Furthermore, the fundus examination showed notable retinal arteriolar attenuation and subsequent vascular sheathing, which were consistent with previously reported CADASIL-related retinal manifestations (21).

Therefore, the key finding is that presentation of NAION and retinal hypoperfusion in a young patient without any other risk factors necessitates consideration of secondary causes. These manifestations could represent a potential presentation of CADASIL.

## Conclusion

In conclusion, our case indicates that visual disturbances can serve as the initial manifestation of CADASIL. These include NAION and macular alterations secondary to retinal hypoperfusion. It is crucial to heighten awareness of these atypical presentations, particularly in young patients without significant basic diseases. This increased awareness is essential to facilitating early diagnosis and management in patients with this inherited cerebral small vessel disease. On the other hand, given that the possibility of visual function impairment caused by CADASIL has been clearly identified, monitoring retinal blood vessels is extremely important for CADASIL patients. This can help detect problems in a timely manner, initiate treatment as soon as possible, and improve the prognosis of vision.

## Data Availability

The original contributions presented in the study are included in the article/supplementary material, further inquiries can be directed to the corresponding author.
